# Rupture of Left Kidney

**Published:** 1882-10

**Authors:** R. N. Isham


### ARTICLE IV.

RUPTURE OF LEFT KIDNEY. Report of a Case, with Autopsy. By R. N. ISHAM, M.D.

July 15, 11:30 A. M., Ed. Rillen, while at work on a locomotive, slipped and fell from the gangway through a clear space of four feet, striking upon his left side across a wooden horse. I saw him at 2 P. M., nearly three hours afterward, and found him in a condition bordering upon collapse, as if from internal hæmorrhage. Countenance pallid; expression anxious; pain referred to the region of the diaphragm; surface of the body covered with a cold perspiration, and pulse hardly perceptible at the wrist. No visible sign of injury on inspection. Percussion gave an area of dullness in the right hypochondriac region, over a larger extent of territory than natural, and on drawing off the urine, it presented characteristic appearance of hæmatine, being of a smoky brownish color. Reaction was accomplished in a few hours, and without any continuance of the hæmaturia. The case progressed favorably for five days, till convalescence was apparently fairly established. During that interval of quiet there was but little pain, no febrile excitement, no disturbance of the stomach, and no sign of peritoneal injury.

On the morning of the ninth day, after a movement of the bowels and considerable exercise on the preceding day, he was suddenly seized with the most violent paroxysms of pain, accompanied by loud cries, and referred to the lower portion of the abdomen and cord. I found him again presenting signs of collapse, with incessant vomiting of a greenish fluid, and supporting the scrotum; he was positive of a rupture. Chloroform was administered, and the examination made of the patient showed an infiltration or dissection of the infundibuliform process with blood, reaching to the scrotum or tunica vaginalis. Considerable fullness in the left hypochondriac and lumbar region, could be recognized by palpation and percussion.

From this time, during the following days, the symptoms were those of peritonitis, at first circumscribed, then general; a thready, quick pulse and heightened temperature until the morning of the 20th, when he suddenly expired, having only a few hours previously expressed himself as much improved and feeling better.

Recognizing the character of the injury as laceration of the kidney on my first visit, I believed that a recurrence of the hæmorrhage had taken place on the occasion of the relapse of the symptoms; and, on the afternoon of the 19th, I made a tentative exploration of the left lumbar region with a small needle and syringe, and found that I was able to evacuate liquid blood, but using an aspirator with a larger needle, I was only able to withdraw about four oz. of blood, with fragments of clots, which satisfied me that the effusion was coagulated. I had refrained from any operative interference, because the attendants had noticed a fæcal odor to the vomited matters (as they supposed), and the persistent vomiting after the relapse suggested to me the probability of a contused, strictured and gangrenous intestine.

Autopsy July 20, 3 P. M. Body five feet eight inches in height, weighed 136 pounds. No mark or discoloration on the body. Abdominal section showed the intestine moderately distended by gas, and discolored through the mesentery by sanguineous injection It presented no other lesion, and, after its removal, a large distended, circumscribed collection of blood was discovered in the right lumbar region. Behind the peritoneum there was a collection of six to ten oz. of sero-purulent fluid. This collection of blood was dark-colored, coagulated, and contained several concentric layers of partially organized fibrinous clots, as is represented by aneurismal sacs. In this was found the remnants of the kidney, as I now show you, absolutely rent and smashed in three parts, and two-thirds of it disintegrated and almost unrecognizable, except its capsule—so intimately blended was it with the coagula by dissection of the hæmorrhage. There were evidences of gangrene progressing in the peritoneum and subperitoneal tissues, extending to the pelvic cavity and over the bladder.

There was a singular absence in this case of the sign of ruptured kidney so generally spoken of—that of a frequent desire to void the urine, which can only be explained by reason of the most complete destruction of the gland by the injury, and the complete isolation of it by the effused blood during the first few days after the reception of the injury.

The most reliable sign of laceration of the kidney, taken in connection with the external symptoms, is that of the hæmaturia ; but in some cases this may not be present or may not be recognized. Thus, Erichsen mentions a case where the kidney was completely smashed, as in this one, without any appearance of this sign, no bloody urine having found its way into the bladder ; still, the recurrence of this sign, in connection with an injury competent to rupture this organ, may be assumed to be pathognomonic, and its appearance should always be looked for as an aid in the diagnosis of lumbar injuries of severity—even to the resort, if necessary, to catheterization. In this case, there was enough urine secreted by the mutilated remains of the kidney to cause gangrene and sero-purulent effusion.

Laceration of the kidney is a regularly fatal accident, and chiefly due to the extravasation of urine which occurs when the capsule is torn. Of sixteen cases collected by myself, but one recovery took place, and in this instance, a case of gun-shot injury, a bit of clothing was wedged in the gland, and came away after many months. During this time more or less urine escaped through the fistula. The wound healed at once. The foreign body escaped. The other cases were free from hæmorrhage, gangrene and suppuration, and death occurred in six to fourteen days.

I am convinced that in these cases a provision should be made for drainage, and in cases of destructive laceration, as would be indicated by excessive hæmorrhage, removal of the entire remnant of the gland be performed. The case of recovery just cited shows the good effects of a provision for drainage, and this which I have reported fully establishes the fact that if the mutilated organ had been removed, the patient might have recovered; or, at least, the only chance of recovery would have been offered to him by so doing.

The first case of removal for injury, I find was by Bremdt (Vienna) in 1873 ; that of a young man stabbed with a knife, operated upon four days after the injury, followed by recovery. Inasmuch as this operation—removal of one kidney—presuming the remaining one to be sound, is very successful; that the approach to the kidney is safe and easy; that in exploratory incisions the peritoneal sac need not be disturbed, I am further convinced that this procedure should be included in the measures of treatment of such cases.

